# Supplementary material for: Predicting the pathway involvement of metabolites annotated in the MetaCyc knowledgebase
Source: BMC Bioinformatics. 2026 Jan 7;27:36. doi: 10.1186/s12859-025-06358-z (PMC12870939; doi:10.1186/s12859-025-06358-z)
Supplement: Supplementary file 3 — Supplementary Material 3 [file 12859_2025_6358_MOESM3_ESM.docx]

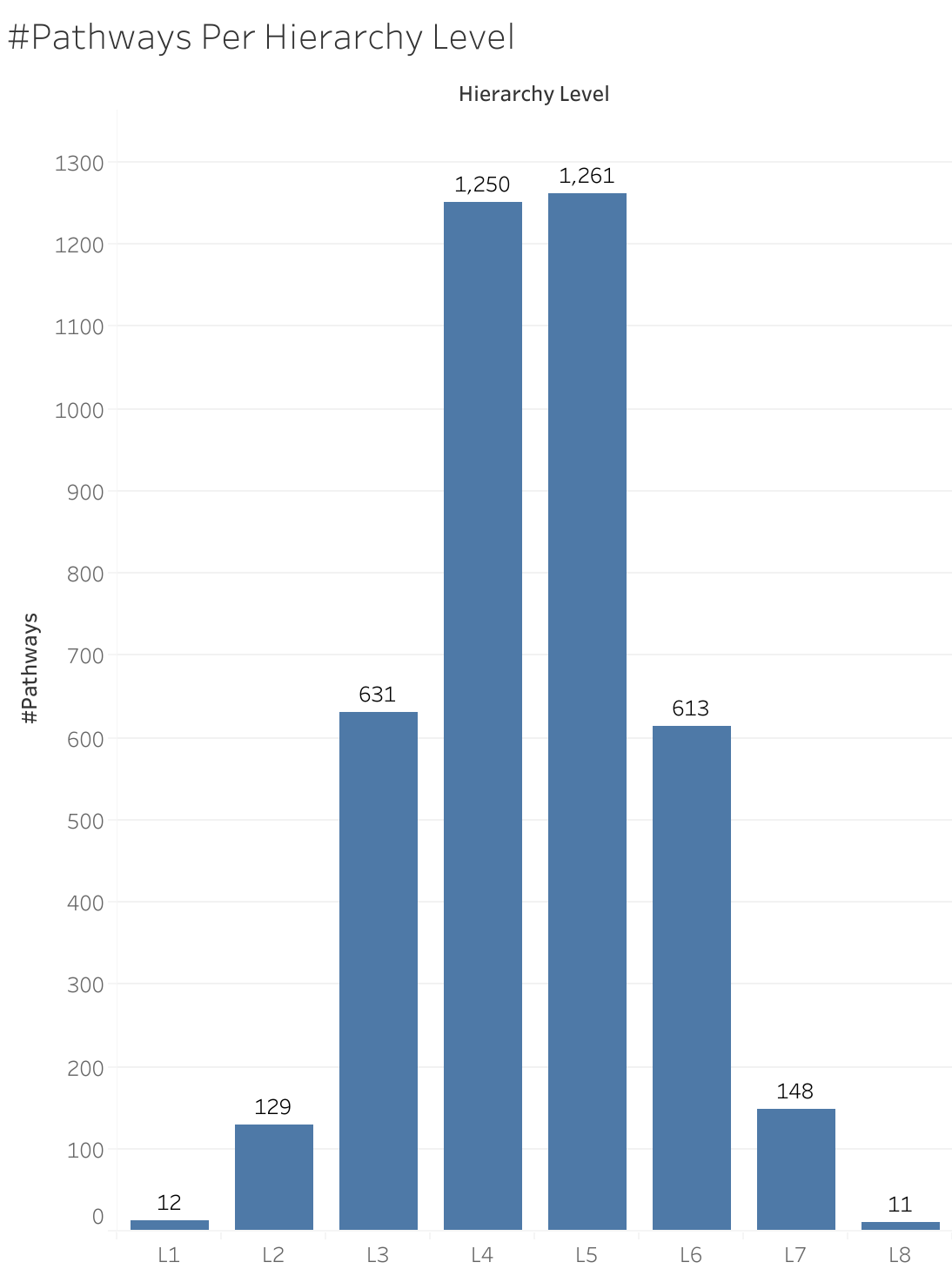


Fig 1 - The number of pathways within each hierarchy level.

Table 1 - Details related to the proportion of positive entries in the train sets and test sets of every cross-validation iteration for each dataset.

| **Dataset** | **Split** | **Total #Entries** | **#Positive Entries** | **Positive Proportion** |
| --- | --- | --- | --- | --- |
| **L1+** | Train | 35,936,626 | 117,496 | 0.32695% |
|  | Test | 3,992,959 | 1,3055 | 0.32695% |
| **L2+** | Train | 35,830,278 | 102,163 | 0.28513% |
|  | Test | 3,981,143 | 11,351 | 0.28512% |
| **L3+** | Train | 34,687,042 | 83,346 | 0.24028% |
|  | Test | 3,854,116 | 9,261 | 0.24029% |

Table 2 - Hyperparameter values resulting from the hyperparameter tuning algorithm.

| **Hyperparameter** | **Value** |
| --- | --- |
| **Activation Function** | RReLU |
| **Hidden Dimension Size** | 1292 |
| **Number of Layers** | 5 |
| **Dropout** | 0.020310844777206800 |
| **Learning Rate** | 0.0005931305104533710 |
| **Beta1** | 0.44931561705366900 |
| **Beta2** | 0.36183360331299900 |
| **EPS** | 0.0007418554873244980 |
| **Weight Decay** | 3.42685823734844E-05 |
| **Classification Threshold** | 0.6565705465951340 |

*Table 3 – Statistics of performance metrics for models trained on the L1+, L2+, and L3+ datasets.*

| **Pathway Hierarchy Levels Included** | **Metric** | **Mean Score** | **Median Score** | **Standard Deviation** |
| --- | --- | --- | --- | --- |
| **L1+** | Accuracy | 0.999 | 0.999 | 0.0001 |
|  | F1 Score | 0.840 | 0.841 | 0.0113 |
|  | MCC | 0.845 | 0.845 | 0.0101 |
|  | Precision | 0.759 | 0.762 | 0.0195 |
|  | Recall | 0.941 | 0.941 | 0.0064 |
|  | Specificity | 0.999 | 0.999 | 0.0001 |
| **L2+** | Accuracy | 0.999 | 0.999 | 0.0001 |
|  | F1 Score | 0.829 | 0.830 | 0.0130 |
|  | MCC | 0.834 | 0.835 | 0.0113 |
|  | Precision | 0.746 | 0.746 | 0.0250 |
|  | Recall | 0.933 | 0.934 | 0.0079 |
|  | Specificity | 0.999 | 0.999 | 0.0001 |
| **L3+** | Accuracy | 0.999 | 0.999 | 0.0001 |
|  | F1 Score | 0.815 | 0.816 | 0.0093 |
|  | MCC | 0.820 | 0.821 | 0.0080 |
|  | Precision | 0.730 | 0.734 | 0.0172 |
|  | Recall | 0.922 | 0.921 | 0.0072 |
|  | Specificity | 0.999 | 0.999 | 0.0001 |


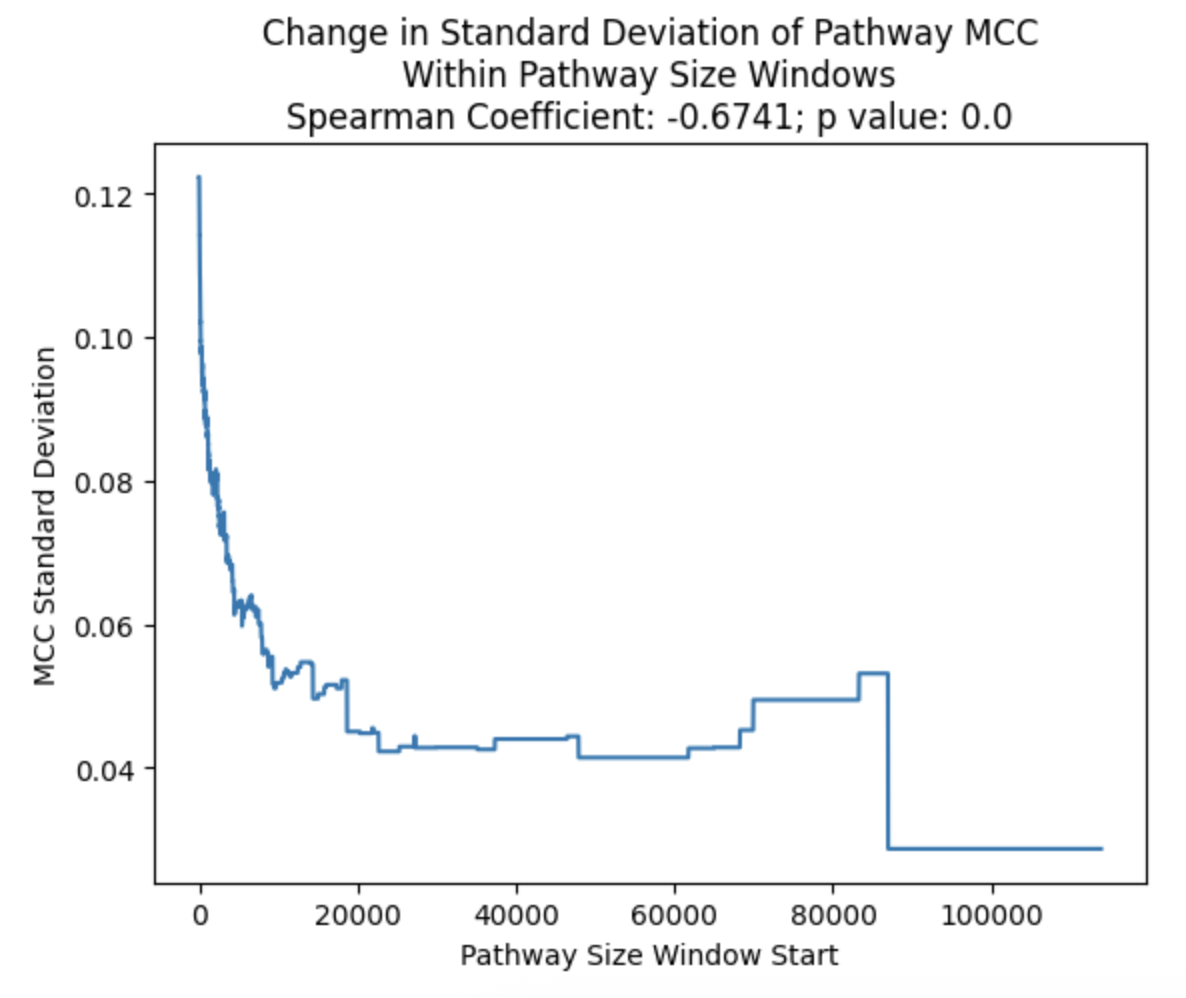


Fig 2 -Sliding window analysis demonstrating the trend downwards of pathway MCC standard deviation as the pathway size increases.
